# Anterior cervical discectomy and fusion versus anterior cervical corpectomy and fusion in multilevel cervical spondylotic myelopathy: A meta-analysis

**DOI:** 10.1097/MD.0000000000005437

**Published:** 2016-12-09

**Authors:** Tao Wang, Hui Wang, Sen Liu, Huang-Da An, Huan Liu, Wen-Yuan Ding

**Affiliations:** a Department of Spinal Surgery, The Third Hospital of Hebei Medical University; b Hebei Provincial Key Laboratory of Orthopedic Biomechanics, Shijiazhuang, China.

**Keywords:** anterior cervical corpectomy and fusion, anterior cervical discectomy and fusion, clinical outcomes, multilevel cervical spondylotic myelopathy, radiographic outcomes, surgical outcomes

## Abstract

**Background:**

Both anterior cervical discectomy and fusion (ACDF) and anterior cervical corpectomy and fusion (ACCF) are used to treat multilevel cervical spondylotic myelopathy (mCSM); however, which one is better treatment for mCSM remains considerable controversy. A meta-analysis was performed to compare clinical outcomes, radiographic outcomes, and surgical outcomes between ACDF and ACCF in treatment for mCSM.

**Methods:**

An extensive search of literature was performed in Pubmed/MEDLINE, Embase, the Cochrane library, CNKI, and WANFANG databases on ACDF versus ACCF treatment for mCSM from January 2011 to August 2016. The following variables were extracted: length of hospital stay, blood loss, operation time, Japanese Orthopedic Association (JOA) scores, Neck Disability Index (NDI) score, fusion rate, Cobb angles of C2 to C7, dysphagia, hoarseness, C5 palsy, infection, cerebral fluid leakage, donor site pain, epidural hematoma, graft subsidence, graft dislodgment, pseudoarthrosis, and total complications. Data analysis was conducted with RevMan 5.3 and STATA 12.0.

**Results:**

A total of 8 studies containing 878 patients were included in our study. The results showed that ACDF is better than ACCF in the angle of C2 to C7 at the final follow-up (*P* < 0.00001, standardized mean difference = 4.76 [3.48, 6.03]; heterogeneity: *P* = 0.17, *I*^2^ = 43%), C5 plasy (*P* = 0.02, odds ratio [OR] 0.42, 95% confidence interval [CI] 0.21, 0.86; heterogeneity: *P* = 0.52, *I*^2^ = 0%), blood loss (*P* < 0.00001, standardized mean difference = −53.12, 95% CI −64.61, −41.64; heterogeneity: *P* = 0.29, *I*^2^ = 20%), fusion rate (*P* = 0.04, OR 2.54, 95% CI 1.05, 6.11; heterogeneity: *P* = 0.29, *I*^2^ = 20%), graft subsidence (*P* = 0.004, OR 0.11, 95% CI 0.02, 0.48; heterogeneity: *P* = 0.94, *I*^2^ = 0%), and total complications (*P* = 0.0009, OR 0.56, 95% CI 0.40, 0.79; heterogeneity: *P* = 0.29, *I*^2^ = 18%).However, there are no significant differences in length of hospital stay, operation time, JOA scores, NDI scores, preoperative angle of C2 to C7, dysphagia, hoarseness, infection, cerebral fluid leakage, donor site pain, epidural hematoma, graft dislodgment, and pseudoarthrosis (all *P* > 0.05).

**Conclusions:**

Based on our meta-analysis, our results suggest that both ACDF and ACCF are good plans in clinical outcomes; however, ACDF is a better choice in radiographic outcomes and total complications for the treatment of multilevel CSM.

## 1 Introduction

Cervical spondylotic myelopathy (CSM), a common clinical degenerative disease, seriously influences quality of life and even leads to disability for the old population.^[1–3]^ CSM is usually caused by narrowing of the cervical spinal canal due to degenerative and congenital changes.^[3–6]^ The selection of optimal surgical treatment for CSM, especially for multilevel cervical spondylotic myelopathy (mCSM), remains controversial.^[1–9]^ Surgeries mainly involved anterior and posterior approaches, including anterior cervical discectomy and fusion (ACDF),^[10–12]^ anterior cervical corpectomy and fusion (ACCF),^[11–15]^ laminoplasty,^[16–20]^ laminectomy,^[12,18–22]^ and laminectomy with fusion.^[20–23]^ ACDF for treating CSM was firstly introduced by Smith and Robinson^[24]^ and Cloward^[25]^; the anterior procedure has become the most widely used surgical choice.^[26]^ Among the anterior approaches, ACDF can decompress the anterior spinal cord and preserve the stability of the spinal column^[27–33]^; however, ACDF may have a high risk of incomplete decompression, limited visual exposure, and injury to the cord.^[30–36]^ ACCF also provides a more extensive decompression and serves as a source for autografting.^[37–41]^ Unfortunately, ACCF is a more difficult spinal surgery to perform and also has a higher incidence of complications, such as injury to the spinal cord or nerve roots, excessive bleeding, graft displacement, or extrusion.^[40–45]^

Previous meta-analyses^[46,47]^ reviewed mainly focused on the comparison between ACDF and ACCF for 1-level or 2-level CSM, few variables, or included studies from 1980s or1990s. However, the clinical efficacy and complications of ACDF compared with ACCF in patients with mCSM still remain controversial. The purpose of this meta-analysis is to compare clinical outcomes, radiographic outcomes, and surgical outcomes of ACDF compared with ACCF in treatment for mCSM.

## 2 Materials and methods

### 2.1 Ethics statement

There is no need to seek informed consent from patients, since this is a meta-analysis based on the published data, without any potential harm to the patients; this is approved by Ethics Committee of The Third Hospital of HeBei Medical University.

### 2.2 Search strategy

An extensive search of literature was performed in PubMed, Embase, the Cochrane library, CNKI, and WANFANG databases. The following key words were used for search: “anterior cervical discectomy and fusion,”, “anterior cervical corpectomy and fusion,” “multilevel cervical spondylotic myelopathy,” from January 2011 to August 2016, with various combinations of the operators “AND” and “OR”. Language was restricted to Chinese and English.

### 2.3 Inclusion criteria

Studies were included if they met the following criteria: randomized or nonrandomized controlled study; age greater than or equal to 18 years; studies compared ACDF with ACCF for treatment of CSM; 3 or 4 levels cervical spondylotic myelopathy; follow-up more than 2 years.

### 2.4 Exclusion criteria

Studies were excluded if they met the following criteria: dealt only with combined ACDF and ACCF surgery versus ACDF or ACCF alone for treatment of CSM; had an average follow-up time of less than 2 years; had repeated data; did not report outcomes of interest; in vitro human cadaveric biomechanical studies; earlier trial, reviews, and case-reports; have ossification of posterior longitudinal ligament.

### 2.5 Selection of studies

Two reviewers independently reviewed all subjects, abstracts, and the full text of articles. Then the eligible trials were selected according to the inclusion criteria. When consensus could not be reached, a third reviewer was consulted to resolve the disagreement.

### 2.6 Data extraction and management

Two reviewers extracted data independently. The data extracted included the following categories: study ID; study design; study location; total patients; follow-up; mean age; sex, clinical outcomes—length of hospital stay, preoperative and the final follow-up Japanese Orthopedic Association (JOA) scores, preoperative and the final follow-up Neck Disability Index (NDI) scores; radiographic outcomes—preoperative and the final follow-up Cobb angles of C2 to C7, fusion rate, graft subsidence, graft dislodgment; and surgical outcomes—blood loss, operation time, dysphagia, hoarseness, C5 palsy, infection, cerebral fluid leakage, donor site pain, epidural hematoma, and pseudoarthrosis.

### 2.7 Statistical analysis

We analyzed data by RevMan 5.3 (The Nordic Cochrane Center, The Cochrane Collaboration, Copenhagen, Denmark) and STATA 12.0 (Stata Corporation, College Station, TX). Odds ratio (OR), as a summary statistic, was applied to analyze dichotomous variables, and continuous variables were analyzed by standardized mean difference (SMD). Both were reported with 95% confidence intervals (CIs), and *P* value <0.05 presented statistical significance. We assessed statistical heterogeneity by the *I*^2^ tests, which described the proportion of the total variation from 0% to 100% in meta-analysis assessments. If *I*^2^ was >50%, which implies an obvious heterogeneity, we chose random-effects model using for the analysis If *I*^2^ was ≤50%, which implies no significant heterogeneity, fixed-effects model was used to analyze.^[48,49]^

### 2.8 Test for risk of publication bias

Funnel plot as a visual inspection was used to assess publication bias. If there is publication bias, funnel plot should be asymmetric, but if there is no publication bias, funnel plot should be symmetric. Egger and Begg tests were used to evaluate the funnel plot asymmetry; if *P* < 0.05, we considered it as a significance level.

## 3 Results

### 3.1 Search results

**Figure 1:**
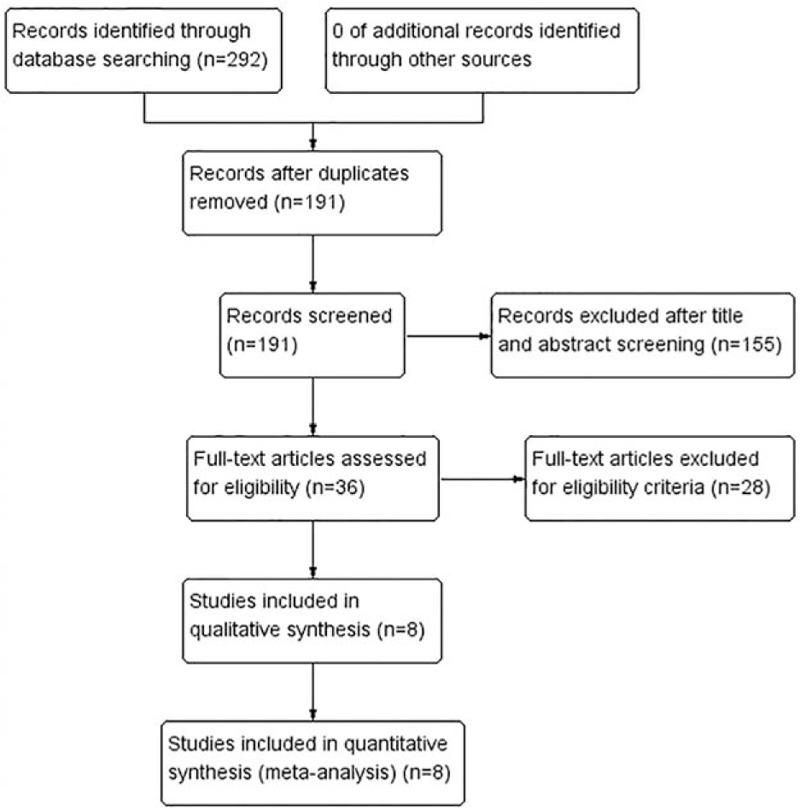
Flow diagram of study selection.

We had searched 206 English studies in MEDLINE and Embase, and 86 Chinese studies in WANFANG and CNKI databases. Of these, 50 English articles and 51 Chinese articles after duplicates were removed; 128 English articles and 27 Chinese articles were excluded due to unrelated studies. Twenty-two English articles and 6 Chinese articles were excluded due to eligibility criteria. As a result, a total of 8 studies were identified for this meta-analysis. The literature search procedure is shown in Fig. 1.

### 3.2 Baseline characteristics and quality assessment

In all, 878 patients with mCSM from 8 studies were included in our study. Table 1 shows the baseline characteristics of included articles.

**Table 1:**
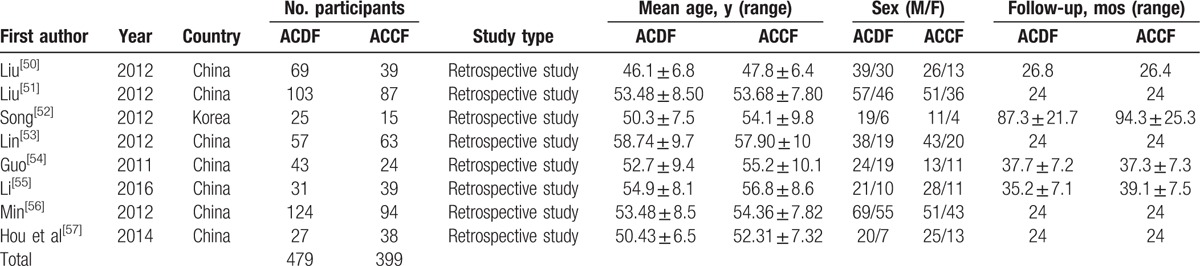
Characteristics of included studies.

All included studies were retrospective studies. Newcastle Ottawa Quality Assessment Scale (NOQAS) was applied to estimate the quality of each study. We used NOQAS, the maximum of 9 points, to assess quality of selection for nonrandomized case-controlled studies and cohort studies in term of comparability, exposure, and outcomes. Among these studies, 5 studies scored 8 points and 3 studies scored 7 points. Therefore, each study had relatively high quality (Table 2).

**Table 2:**
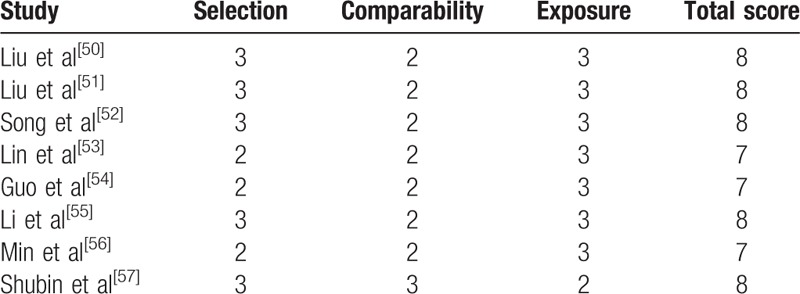
The quality assessment according to the Newcastle Ottawa Quality Assessment Scale (NOQAS) of each study.

### 3.3 Clinical outcomes

#### 3.3.1 JOA score

**Figure 2:**
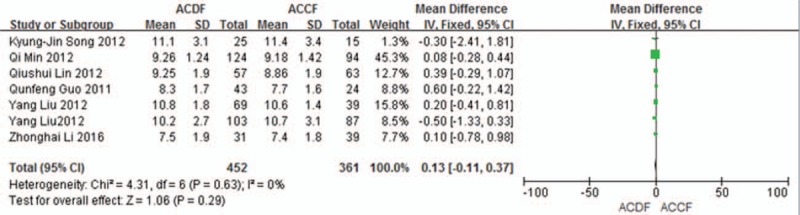
The standardized mean difference (SMD) estimate preoperative JOA score in 2 groups. CI = confidence interval, df = degrees of freedom, JOA = Japanese Orthopedic Association, M-H = Mantel–Haenszel.

**Figure 3:**
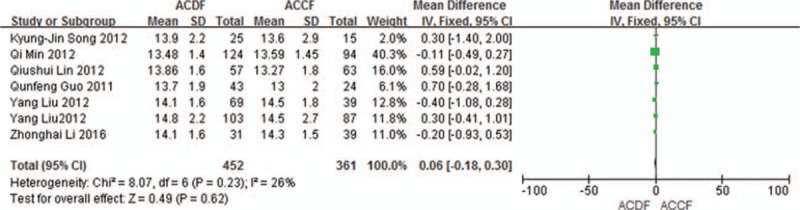
Forest plot showing at the final follow-up JOA score in 2 groups. CI = confidence interval, df = degrees of freedom, JOA = Japanese Orthopedic Association, M-H = Mantel–Haenszel.

Seven studies (813 of 878 patients)^[50–56]^ reported preoperative and the final follow-up JOA scores between ACDF and ACCF. The meta-analysis showed that there is no significant difference between ACDF and ACCF in preoperative and the final follow-up JOA scores (*P* = 0.29, SMD = 0.13 [−0.11, 0.37]; heterogeneity: *P* = 0.63, *I*^2^ = 0%, fixed-effects model, Fig. 2; *P* = 0.62, SMD = 0.06 [−0.18, 0.30]; heterogeneity: *P* = 0.23, *I*^2^ = 26%, fixed-effects model, Fig. 3).

#### 3.3.2 NDI score

**Figure 4:**
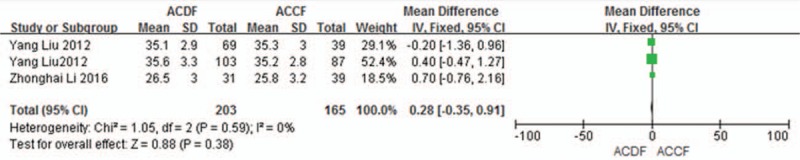
The standardized mean difference (SMD) estimate preoperative NDI score in 2 groups. CI = confidence interval, df = degrees of freedom, M-H = Mantel–Haenszel, NDI = Neck Disability Index.

**Figure 5:**
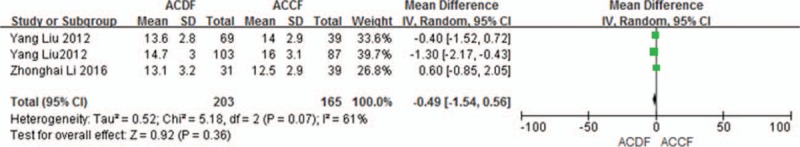
The standardized mean difference (SMD) estimate at the final follow-up NDI score in 2 groups. CI = confidence interval, df = degrees of freedom, M-H = Mantel–Haenszel, NDI = Neck Disability Index.

Three studies (368 of 878 patients)^[50,51,55]^ reported preoperative and the final follow-up NDI scores between ACDF and ACCF. The meta-analysis showed that there is no significant difference between ACDF and ACCF in preoperative and the final follow-up NDI scores (*P* = 0.38, SMD = 0.28 [−0.35, 0.91]; heterogeneity: *P* = 0.59, *I*^2^ = 0%, fixed-effects model, Fig. 4; *P* = 0.36, SMD = −0.49 [−1.54, 0.56]; heterogeneity: *P* = 0.07, *I*^2^ = 61%, random-effects model, Fig. 5).

#### 3.3.3 Hospital stay

**Figure 6:**
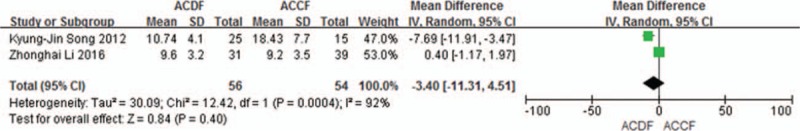
The standardized mean difference (SMD) estimate hospital stay in 2 groups. CI = confidence interval, df = degrees of freedom, M-H = Mantel–Haenszel.

Two studies (110 of 878 patients)^[52,55]^ reported hospital stay between ACDF and ACCF. The meta-analysis showed that there is no significant difference between ACDF and ACCF in hospital stay (P = 0.40, SMD = −3.40 [−11.31, 4.51]; heterogeneity: *P* = 0.00004, *I*^2^ = 92%, random-effects model, Fig. 6).

### 3.4 Radiographic outcomes

#### 3.4.1 The angle of C2 to C7

**Figure 7:**
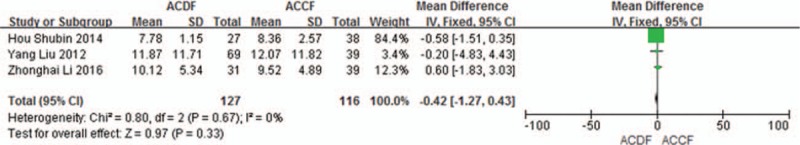
The standardized mean difference (SMD) estimate preoperative the angle of C2 to C7 in 2 groups. CI = confidence interval, df = degrees of freedom, M-H = Mantel–Haenszel.

**Figure 8:**
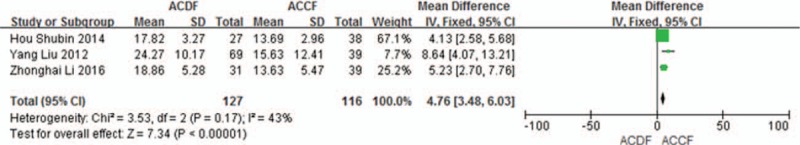
The standardized mean difference (SMD) estimate at the final follow-up of the angle of C2 to C7 in 2 groups. CI = confidence interval, df = degrees of freedom, M-H = Mantel–Haenszel.

Three studies (243 of 878 patients)^[50,55,57]^ reported preoperative and the final follow-up angle of C2 to C7 between ACDF and ACCF. The meta-analysis showed that there is no difference between ACDF and ACCF in preoperative angle of C2 to C7 (*P* = 0.33, SMD = −0.42 [−1.27, 0.43]; heterogeneity: *P* = 0.67, *I*^2^ = 0%, fixed-effects model, Fig. 7), but significant difference in the final follow-up angle of C2 to C7 (*P* < 0.00001, SMD = 4.76 [3.48, 6.03]; heterogeneity: *P* = 0.17, *I*^2^ = 43%, fixed-effects model, Fig. 8).

#### 3.4.2 Fusion rate

**Figure 9:**
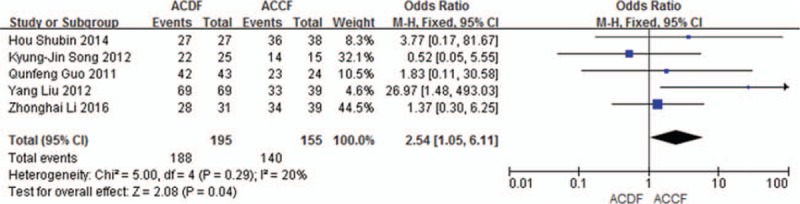
Forest plot showing fusion rate in 2 groups. CI = confidence interval, df = degrees of freedom, M-H = Mantel–Haenszel.

Five studies (350 of 878 patients)^[50,52,54–55,57]^ reported fusion rate between ACDF and ACCF. The meta-analysis showed that ACDF have a better result of fusion rate than that of ACCF (*P* = 0.04, OR 2.54, 95% CI 1.05, 6.11; heterogeneity: *P* = 0.29, *I*^2^ = 20%, fixed-effects model, Fig. 9).

#### 3.4.3 Graft subsidence

**Figure 10:**
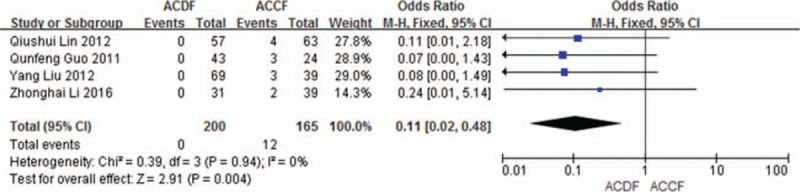
Forest plot showing graft subsidence in 2 groups. CI = confidence interval, df = degrees of freedom, M-H = Mantel–Haenszel.

Four studies (365 of 878 patients)^[50,53–55]^ reported incidence of graft subsidence between ACDF and ACCF. The meta-analysis showed that ACDF is less than ACCF in incidence of graft subsidence (*P* = 0.004, OR 0.11, 95% CI 0.02, 0.48; heterogeneity: *P* = 0.94, *I*^2^ = 0%, fixed-effects model, Fig. 10).

#### 3.4.4 Graft dislodgment

**Figure 11:**
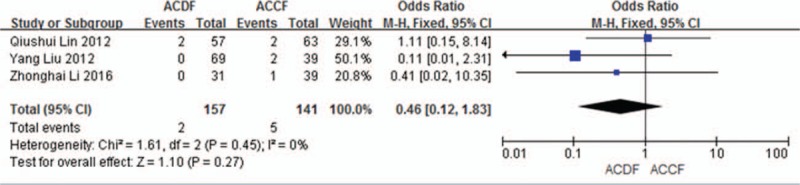
Forest plot showing graft dislodgment in 2 groups. CI = confidence interval, df = degrees of freedom, M-H = Mantel–Haenszel.

Three studies (298 of 878 patients)^[50,53,55]^ reported incidence of graft dislodgment between ACDF and ACCF. The meta-analysis showed that there is no significant difference (*P* = 0.27, OR 0.46, 95% CI 0.12, 1.83; heterogeneity: *P* = 0.45, *I*^2^ = 0%, fixed-effects model, Fig. 11).

### 3.5 Surgical outcomes

#### 3.5.1 Blood loss

**Figure 12:**
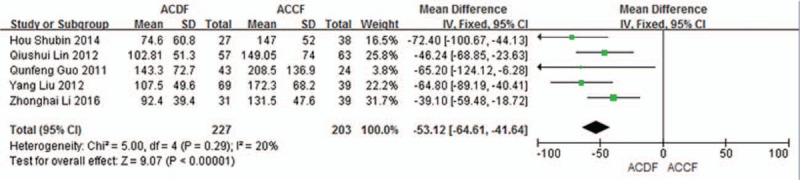
The standardized mean difference (SMD) estimate blood loss in 2 groups. CI = confidence interval, df = degrees of freedom, M-H = Mantel–Haenszel.

Five studies (430 of 878 patients)^[50,53–55,57]^ reported blood loss between ACDF and ACCF. The meta-analysis showed that ACDF is less than ACCF in blood loss (*P* < 0.00001, SMD = −53.12 [−64.61, −41.64]; heterogeneity: *P* = 0.29, *I*^2^ = 20%, fixed-effects model, Fig. 12).

#### 3.5.2 Operation time

**Figure 13:**
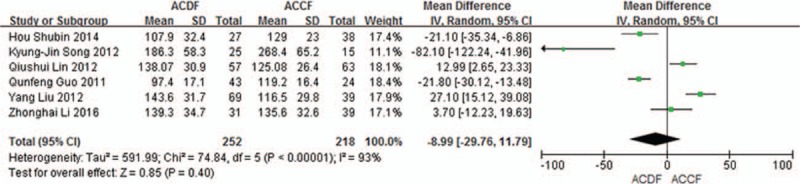
The standardized mean difference (SMD) operation time in 2 groups. CI = confidence interval, df = degrees of freedom, M-H = Mantel–Haenszel.

Six studies (470 of 878 patients)^[50,52–55,57]^ reported operation time between ACDF and ACCF. The meta-analysis showed that there is no significant difference between ACDF and ACCF in operation time [(*P* = 0.40, SMD = −8.99 [−29.76, 11.79]; heterogeneity: *P* < 0.00001, *I*^2^ = 93%, random-effects model, Fig. 13).

#### 3.5.3 Total complications

**Figure 14:**
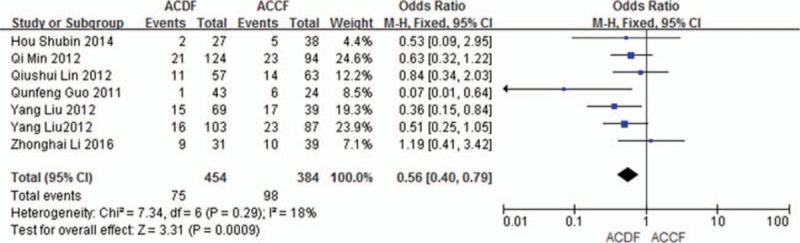
Forest plot showing number of total complications in 2 groups. CI = confidence interval, df = degrees of freedom, M-H = Mantel–Haenszel.

Seven studies (838 of 878 patients)^[50–51,53–57]^ reported incidence of total complications between ACDF and ACCF. The meta-analysis showed that ACDF is less than ACCF in incidence of total complications (*P* = 0.0009, OR 0.56, 95% CI 0.40, 0.79; heterogeneity: *P* = 0.29, *I*^2^ = 18%, fixed-effects model, Fig. 14).

#### 3.5.4 C5 plasy

**Figure 15:**
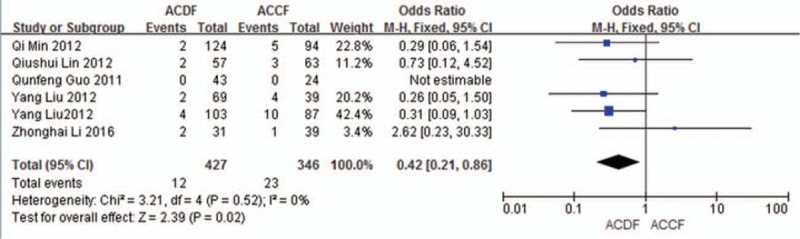
Forest plot showing C5 plasy in 2 groups. CI = confidence interval, df = degrees of freedom, M-H = Mantel–Haenszel.

Six studies (773 of 878 patients)^[50–51,53–56]^ reported incidence of C5 plasy between ACDF and ACCF. The meta-analysis showed that ACDF is less than ACCF in incidence of C5 plasy (*P* = 0.02, OR 0.42, 95% CI 0.21, 0.86; heterogeneity: *P* = 0.52, *I*^2^ = 0%, fixed-effects model, Fig. 15).

#### 3.5.5 Infection

**Figure 16:**
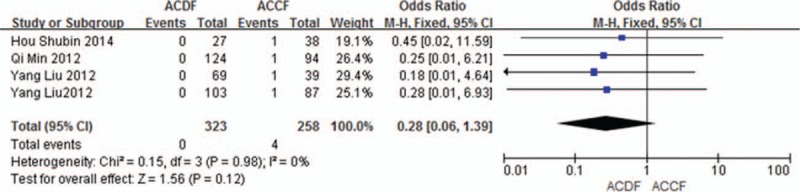
Forest plot showing infection in 2 groups. CI = confidence interval, df = degrees of freedom, M-H = Mantel–Haenszel.

Four studies (581 of 878 patients)^[50–51,56–57]^ reported incidence of infection between ACDF and ACCF. The meta-analysis showed that there is no significant difference (*P* = 0.12, OR 0.28, 95% CI 0.06, 1.39; heterogeneity: *P* = 0.98, *I*^2^ = 0%, fixed-effects model, Fig. 16).

#### 3.5.6 Cerebral fluid leakage

**Figure 17:**
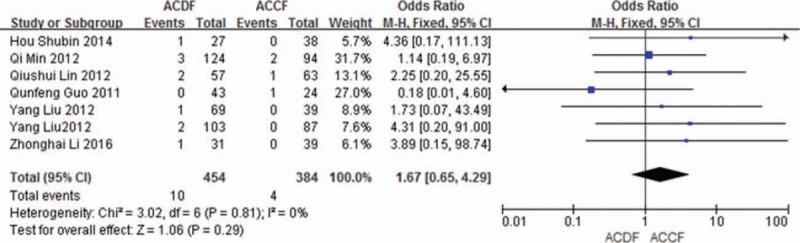
Forest plot showing cerebral fluid leakage in 2 groups. CI = confidence interval, df = degrees of freedom, M-H = Mantel–Haenszel.

Seven studies (838 of 878 patients)^[50–51,53–57]^ reported incidence of cerebral fluid leakage between ACDF and ACCF. The meta-analysis showed that there is no significant difference (*P* = 0.29, OR 1.67, 95% CI 0.65, 4.29; heterogeneity: *P* = 0.81, *I*^2^ = 0%, fixed-effects model, Fig. 17).

#### 3.5.7 Hoarseness

**Figure 18:**
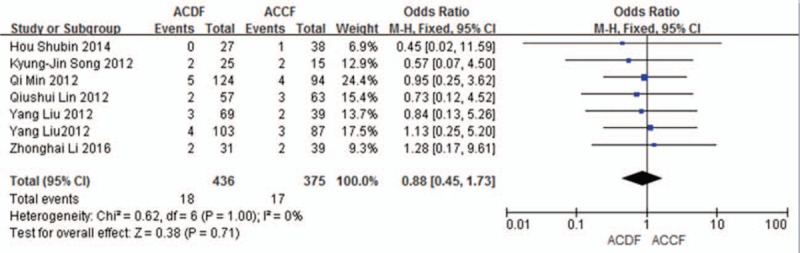
Forest plot showing hoarseness in 2 groups. CI = confidence interval, df = degrees of freedom, M-H = Mantel–Haenszel.

Seven studies (811 of 878 patients)^[50–53,55–57]^ reported incidence of hoarseness between ACDF and ACCF. The meta-analysis showed that there is no significant difference (P = 0.71, OR 0.88, 95% CI 0.45, 1.73; heterogeneity: *P* = 1.00, *I*^2^ = 0%, fixed-effects model, Fig. 18).

#### 3.5.8 Dysphagia

**Figure 19:**
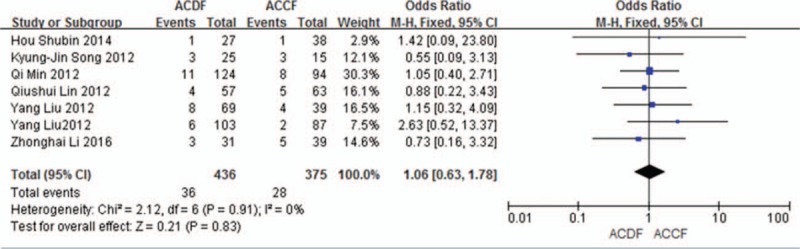
Forest plot showing dysphagia in 2 groups. CI = confidence interval, df = degrees of freedom, M-H = Mantel–Haenszel.

Seven studies (811 of 878 patients)^[50–53,55–57]^ reported incidence of dysphagia between ACDF and ACCF. The meta-analysis showed that there is no significant difference (*P* = 0.83, OR 1.06, 95% CI 0.63, 1.78; heterogeneity: *P* = 0.91, *I*^2^ = 0%, fixed-effects model, Fig. 19).

#### 3.5.9 Epidural hematoma

**Figure 20:**
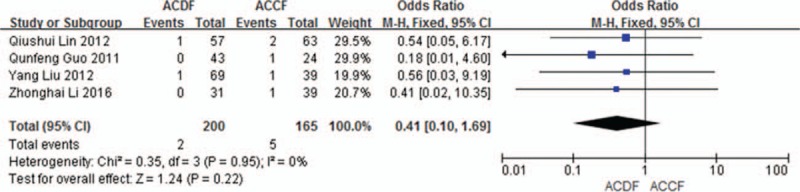
Forest plot showing epidural hematoma in 2 groups. CI = confidence interval, df = degrees of freedom, M-H = Mantel–Haenszel.

Four studies (365 of 878 patients)^[50,53–55]^ reported incidence of epidural hematoma between ACDF and ACCF. The meta-analysis showed that there is no significant difference (P = 0.22, OR 0.41, 95% CI 0.10, 1.69; heterogeneity: *P* = 0.95, *I*^2^ = 0%, fixed-effects model, Fig. 20).

#### 3.5.10 Donor site pain

**Figure 21:**
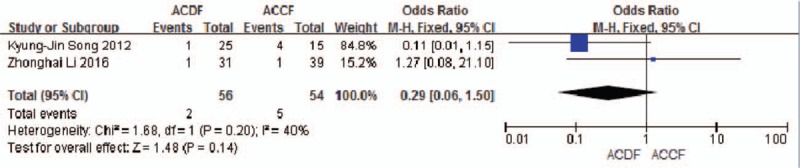
Forest plot showing donor site pain in two groups. CI = confidence interval, df = degrees of freedom, M-H = Mantel–Haenszel.

Two studies (110 of 878 patients)^[52,55]^ reported incidence of donor site pain between ACDF and ACCF. The meta-analysis showed that there is no significant difference (*P* = 0.14, OR 0.29, 95% CI 0.06, 1.50; heterogeneity: *P* = 0.20, *I*^2^ = 40%, fixed-effects model, Fig. 21).

#### 3.5.11 Pseudoarthrosis

**Figure 22:**
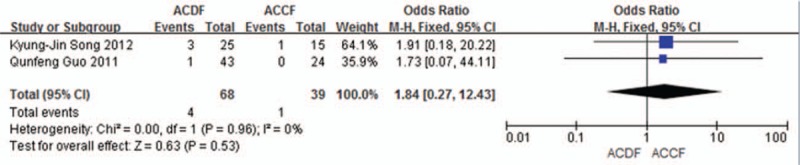
Forest plot showing pseudoarthrosis in 2 groups. CI = confidence interval, df = degrees of freedom, M-H = Mantel–Haenszel.

Two studies (107 of 878 patients)^[52,54]^ reported incidence of pseudoarthrosis between ACDF and ACCF. The meta-analysis showed that there is no significant difference (*P* = 0.53, OR 1.84, 95% CI 0.27, 12.43; heterogeneity: *P* = 0.96, *I*^2^ = 0%, fixed-effects model, Fig. 22).

### 3.6 Publication bias

After a detection of publication bias by STATA 12.0, there was no publication bias found for all included studies (all *P* > 0.05). The funnel plot did not indicate any publication bias in C5 plasy (Begg, *P* = 0.086; Egger, *P* = 0.14); infection (Begg, *P* = 0.734; Egger, *P* = 0.427); pseudoarthrosis (Begg, *P* = 0.296; Egger, *P* = 0.093); cerebral fluid leakage (Begg, *P* = 1.00; Egger, *P* = 0.534); fusion rate (Begg, *P* = 0.296; Egger, *P* = 0.240); graft subsidence (Begg, *P* = 1.00; Egger, *P* = 0.930); graft dislodgment (Begg, *P* = 1.00); hoarseness (Begg, *P* = 1.00); donor site pain (Begg, *P* = 1.00); dysphagia (Begg, *P* = 1.00); total complications (Begg, *P* = 1.00); epidural hematoma (Begg, *P* = 1.00;); the angle of C2 to C7 before surgery (Begg, *P* = 0.296; Egger, *P* = 0.228); the angle of C2 to C7 at final follow-up (Begg, *P* = 0.296; Egger, *P* = 0.228); JOA score before surgery (Begg, *P* = 1.000; Egger, *P* = 0.443); JOA score at final follow-up (Begg, *P* = 0.764; Egger, *P* = 0.723); NDI score before surgery (Begg, *P* = 1.000; Egger, *P* = 0.997); NDI score at final follow-up (Begg, *P* = 0.308; Egger, *P* = 0.619); blood loss (Begg, *P* = 0.462; Egger, *P* = 0.558); operation time (Begg, *P* = 0.06; Egger, *P* = 0.055); hospital stay (Begg, *P* = 1.00).

## 4 Discussion

Recently, some studies^[50–57]^ reported on the surgical plan for mCSM; however, as for mCSM, the option of surgical approach remains debated.^[23,52–55]^ The common operative options included anterior, posterior, and combined anteroposterior approaches. In the 1960s, posterior approaches including laminectomy and laminoplasty were widely used in the treatment of mCSM.^[24–26,58,59]^ But recently the anterior approaches are extensively applied for surgical treatment of mCSM, which can directly decompress the spinal cord and nerve root due to discs herniation or ossification.^[3–7,60]^ Everything has double-edged sword. Complications, such as graft migration, collapse or displacement, hoarseness, dysphagia, C5 palsy, cerebral fluid leakage and infection, of anterior approach are difficult to avoid and these are worth our attention.^[61,62]^

Recently, Liu et al^[50]^ reported the comparison of 3 reconstructive techniques in the treatment for mCSM. In terms of clinical outcomes, radiological parameters, and complication incidence, Liu et al believed that the hybrid surgery (1-level corpectomy plus 1-level discectomy) was the best alternative compared with ACDF and ACCF. Shamji et al^[63]^ reviewed studies on the same topic, but concluded that all 3 operative approaches are effective strategies for the anterior surgical option of mCSM. However, which surgery is a better option in the treatment of mCSM remains unclear. Wen et al^[46]^ and Han et al^[47]^ performed a meta-analysis on comparison of surgical treatment for mCSM between ACDF and ACCF. And they had the same conclusion that both ACDF and ACCF are effective option in treatment for mCSM. Nevertheless, some included studies reported on 1 or 2-level CSM, and some published in 1980s or 1990s influenced accuracy and rigor of the results. So, we collected 8 articles including 878 cases with 3 or 4-level CSM using ACDF and ACCF from January 2011 to August 2016 to compare which one is better for mCSM.

In this meta-analysis, we carried on strict eligibility criteria. Although no randomized controlled trial (RCT) studies were included in our study, all included studies were of high quality according to the NOQAS, and the baseline variables were similar. Thus, we considered the included reports suitable for meta-analysis. We assessed clinical outcomes (length of hospital stay, and JOA and NDI scores), radiographic outcomes (Cobb angles of C2–C7, fusion rate, graft subsidence, and graft dislodgment), and surgical outcomes (blood loss, operation time, dysphagia, hoarseness, C5 palsy, infection, cerebral fluid leakage, donor site pain, epidural hematoma, and pseudoarthrosis) in the meta-analysis. The results showed that there was no marked difference in clinical outcomes (length of hospital stay, and JOA and NDI scores) between ACDF and ACCF. In terms of radiographic outcomes, preoperative Cobb angles of C2 to C7 and incidence of graft dislodgment were similar in the 2 groups. However, in the final follow-up, Cobb angles of C2 to C7, fusion rate, and incidence of graft subsidence ACDF had better results. Although in total complications and blood loss, ACDF are better than these of ACCF, both ACDF and ACCF were similar in operation time, dysphagia, hoarseness, C5 palsy, infection, cerebral fluid leakage, donor site pain, epidural hematoma, and pseudoarthrosis.

In our meta-analysis of preoperative and the final follow-up, JOA and NDI scores were similar in the 2 groups. However, compared with preoperative JOA and NDI, both groups demonstrated a significant increase in final follow-up JOA scores and decrease in final follow-up NDI scores, indicating both ACDF and ACCF can effectively decompress spinal cord by directly removing the anterior pathogenic structures, and in the long term the clinical outcomes were similar in the 2 groups. Besides, in terms of length of hospital stay, the 2 groups were similar, which was different from the result of the study by Han et al.^[47]^ Spinal surgeons can master skillfully surgical techniques, and more than half of Han et al's old included articles may cause difference.

Regarding radiographic outcomes, we found that ACDF and ACCF were similar in preoperative Cobb angles of C2 to C7, and Cobb angles of C2 to C7 at the final follow-up was significantly increased in the 2 groups, but the increase was better in the ACDF group. ACDF can provide more points of distraction and fixation except for the graft and interbody space shaping than these of ACCF. Besides, ACDF can also restore alignment by pulling the involved vertebral bodies toward the lordotic ventral plate.^[22–28,64–66]^ However, ACCF grafts may straighten the cervical spinal column between the remaining vertebral bodies and have fewer force fulcrum, leading to imbalanced force distribution.^[36–42]^ As for graft dislodgment, both the groups had a similar result. Nevertheless, ACDF produced more satisfactory results in incidence of graft subsidence. Obviously, ACDF can offer more fixation points to hold the construct rigidly in place, but ACCF provides only 2 points of fixation, which can explain the reason that more graft-related problems occur in the ACCF group. Previous meta-analyses^[46,47]^ showed that fusion rate between the 2 groups was not significantly different, which is in contrast to our result. Considering some flaws mentioned above in previous meta-analyses,^[46,47]^ we regard the fusion rate was better in ACDF.

We selected blood loss, operation time, and complication-related outcomes to evaluate surgical outcomes and found that ACDF have better results in blood loss, C5plasy, and total complications, whereas other variables including operation time, dysphagia, hoarseness, infection, cerebral fluid leakage, donor site pain, epidural hematoma, and pseudoarthrosis were similar between the 2 groups. C5 palsy is considered as an important complication after cervical decompression surgery. Sakaura et al^[67]^ reported the average incidence was 4.6% (range from 0% to 30%), but pathogenesis of C5 palsy remains unclear till now; multilevel corpectomy may lead to a significant drift of spinal cord away from the ventral side. There were similar rates of dysphagia and hoarseness in both the groups. Dysphagia and hoarseness were common complications after multilevel anterior cervical surgery,^[68]^ which may be caused by trachea and esophagus traction.^[69]^ There is no marked difference in pseudarthrosis, indicating that both ACDF and ACCF can be able to establish a solid arthrodesis and provide inherent mechanical stability of the postdecompressed cervical spine.

There are several limitations of this study. First, there was no RCT comparing the outcomes between ACDF and ACCF; we need RCT for performing further study. Second, the statistical power could be improved in the future by including more studies. Due to the small number of included studies, some parameters could not be analyzed by subgroups to avoid a high heterogeneity, which may exert instability on the consistency of the outcomes. Third, the follow-up of all included article was up to 2 years, which was not enough to observe the long-term recovery and complications. Fourth, the searching strategy was restricted to articles published in English and Chinese languages. Articles with potentially high-quality data that were published in other languages were not included because of anticipated difficulties in obtaining accurate medical translations.

In summary, our meta-analysis showed that both ACDF and ACCF for multilevel CSM have effective results in clinical outcomes (length of hospital stay, and JOA and NDI scores). Because ACDF offers more fixation points and ACCF provides only 2 points of fixation, making ACDF hold the construct rigidly in place. So in radiographic outcomes (at the final follow-up Cobb angles of C2 to C7, fusion rate, and graft subsidence), ACDF had more advantages. Although almost single complication was similar between two groups, but in terms of number of total complications, ACDF produced more satisfactory efficacy. Further studies with high methodological quality and long-term follow-up periods are needed to evaluate the 2 procedures for mCSM treatment.
